# Functional and structural characterization of *Staphylococcus aureus* N‐acetylglucosamine 1‐phosphate uridyltransferase (GlmU) reveals a redox‐sensitive acetyltransferase activity

**DOI:** 10.1002/pro.70111

**Published:** 2025-03-27

**Authors:** Jordan L. Pederick, Akhil Kumar, Tara L. Pukala, John B. Bruning

**Affiliations:** ^1^ Institute for Photonics and Advanced Sensing (IPAS), School of Biological Sciences The University of Adelaide Adelaide South Australia Australia; ^2^ Department of Chemistry School of Physical Sciences, North Terrace Campus, The University of Adelaide Adelaide South Australia Australia

**Keywords:** antibiotic target, cell wall biosynthesis, peptidoglycan, thiol‐based redox switch, UDP‐N‐acetylglucosamine

## Abstract

The bifunctional enzyme N‐acetylglucosamine 1‐phosphate uridyltransferase (GlmU) is a promising antibiotic drug target, as it facilitates the biosynthesis of uridine 5'‐diphospho‐N‐acetylglucosamine, an essential precursor of cell wall constituents. We identified that *Staphylococcus aureus* GlmU (*Sa*GlmU), which was previously targeted for inhibitor development, possesses a dual‐cysteine variation (C379/C404) within the acetyltransferase active site. Enzyme assays performed under reducing and non‐reducing conditions revealed that the acetyltransferase activity of *Sa*GlmU is redox‐sensitive, displaying ~15‐fold lower turnover and ~3‐fold higher *K*_M_ value for the acetyl CoA substrate under non‐reducing conditions. This sensitivity was absent in a C379A *Sa*GlmU mutant. Analysis of *Sa*GlmU by mass spectrometry, x‐ray crystallography, and in silico modeling support that C379 and C404 act as a reversible, redox‐sensitive switch by forming a disulfide under non‐reducing conditions that impedes acetyl CoA recognition and turnover. Therefore, we recommend that future in vitro screening and characterization of *Sa*GlmU inhibitors consider both reducing and non‐reducing conditions.

## 1 INTRODUCTION

Antibiotic resistance represents a significant global public health risk which is predicted to increase in prevalence if countermeasures are not implemented (World Health Organization, 2023; Murray et al., 2022). A direct route to managing antibiotic resistance is through the discovery and development of novel antibiotics. Historically, the essential pathway of bacterial cell wall biosynthesis has yielded the greatest number of clinically useful antibiotics, but not all components of this pathway have been effectively targeted to date (Sarkar et al., 2017; Schneider & Sahl, 2010; Zhou et al., 2022). One example is the bifunctional enzyme N‐acetylglucosamine 1‐phosphate uridyltransferase (GlmU) which completes one of the early cytoplasmic steps in the pathway, converting α‐D‐glucosamine 1‐phosphate (GlcN 1‐P) to uridine diphosphate‐N‐acetylglucosamine (UDP‐GlcNAc). This occurs in a two‐step process, with the acetyltransferase activity of GlmU first catalyzing the reaction of acetyl coenzyme A (AcCoA) and GlcN 1‐P to form CoA and N‐acetylglucosamine 1‐phosphate (GlcNAc 1‐P). Subsequently, the uridyltransferase activity of GlmU catalyzes the Mg^2+^‐dependent reaction of UTP with GlcNAc 1‐P to form UDP‐GlcNAc and pyrophosphate. The UDP‐GlcNAc product is then used as a building block for the synthesis of cell wall constituents such as peptidoglycan, lipopolysaccharides, and teichoic acids, making the function of GlmU essential for bacterial survival (Barreteau et al., 2008; Bertani & Ruiz, 2018; Sewell & Brown, 2014). In particular, the acetyltransferase activity of GlmU has garnered attention for inhibitor development as this activity is unique to prokaryotes. The GlmU isozymes of major human‐pathogenic bacteria including *Escherichia coli*, *Mycobacterium tuberculosis*, *Haemophilus influenzae*, *Streptococcus pneumoniae*, and *Staphylococcus aureus* have therefore been explored as targets for the development of acetyltransferase inhibitors (Buurman et al., 2011; Green et al., 2012; Jia et al., 2023; Pereira et al., 2009; Stokes et al., 2012).

**FIGURE 1:**
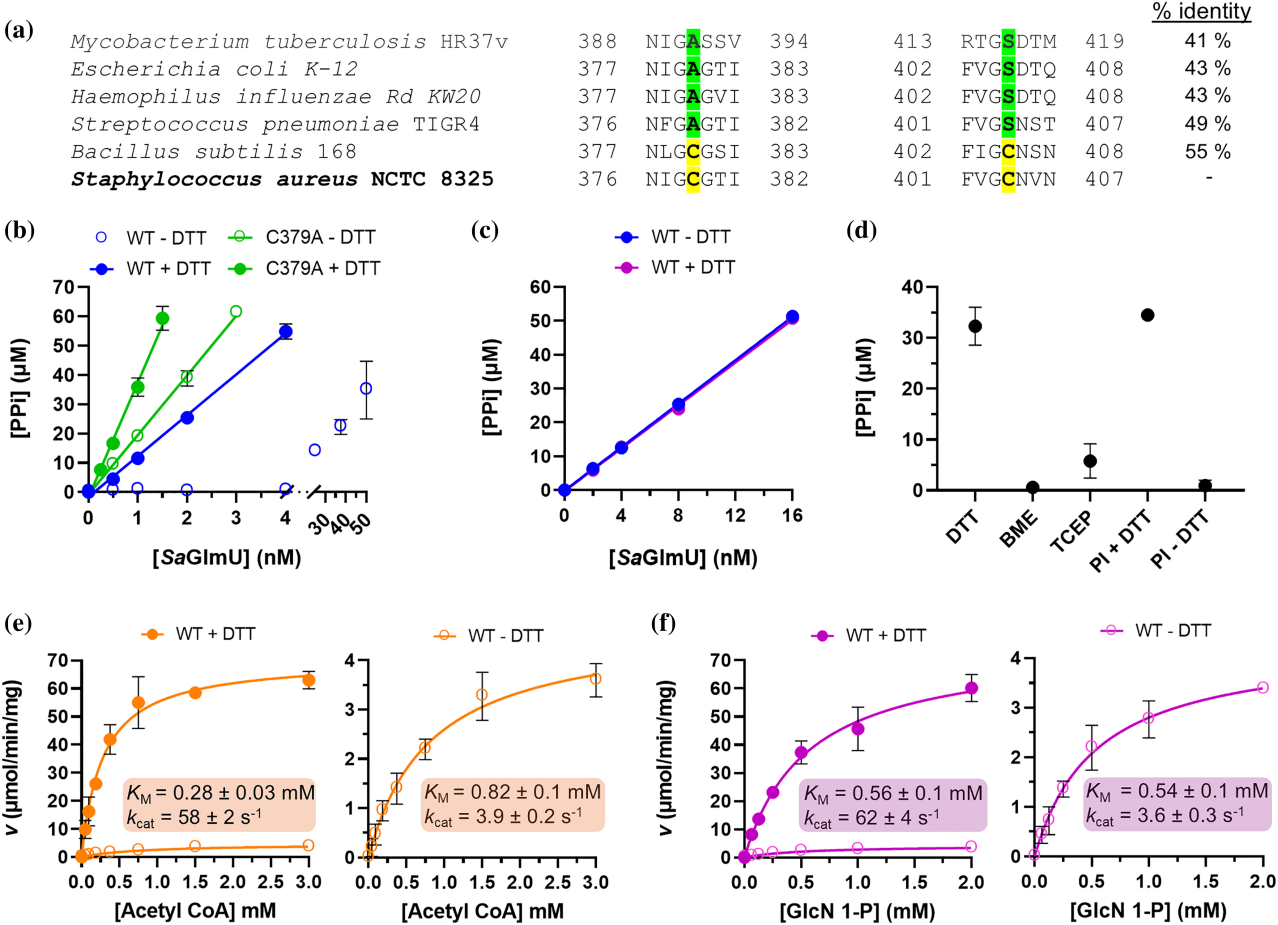
*Sa*GlmU activity is regulated by a reversible redox‐sensitive switch involving C379. (a) Multiple sequence alignment of human pathogenic GlmU isozymes in the region of the dual cysteine variant. The alignment was constructed using ClustalΩ (Thompson et al., 1994). The two positions of interest are denoted by green shading (A/S variant) and yellow shading (C/C variant). Overall amino‐acid sequence identities of each homologue relative to *Sa*GlmU are also provided. (b) Comparison of acetyltransferase activity for WT *Sa*GlmU and C379A *Sa*GlmU with and without 0.5 mM DTT, (c) comparison of uridyltransferase activity for WT *Sa*GlmU with and without 0.5 mM DTT, (d) Effect of reducing agent and DTT preincubation (PI) on the acetyltransferase activity of 2 nM WT *Sa*GlmU, (e) Determination of kinetic parameters for AcCoA with and without 0.5 mM DTT, and (F) Determination of kinetic parameters for GlcN 1‐P with and without 0.5 mM DTT. Data represents the mean ± standard deviation of 3 experiments.

While the structure and function for most of these GlmU isozymes have been characterized in detail, such characterization has not been performed for *S. aureus* GlmU (*Sa*GlmU). On performing a multiple sequence alignment of the *Sa*GlmU acetyltransferase domain to the corresponding region of GlmU isozymes from these human‐pathogenic bacteria, we identified a distinct difference that may influence the acetyltransferase activity of *Sa*GlmU. It was observed that *Sa*GlmU possesses two amino acid substitutions, C379 and C404, which are typically conserved as serine and alanine, respectively (Figure 1a). Previous structural characterization of the closely related *S. pneumoniae* GlmU has revealed that the corresponding residues A379 and S404 are located at the interface of the AcCoA and GlcN 1‐P binding sites, with S404 positioned near the AcCoA substrate (Sulzenbacher et al., 2001). Additionally, it was reported that the GlmU isozyme of the non‐pathogenic bacterium *Bacillus subtilis* (*Bs*GlmU) possesses the same dual‐cysteine substitution (Wang et al., 2017). Kinetic characterization of *Bs*GlmU revealed that the acetyltransferase activity of WT *Bs*GlmU is attenuated under non‐reducing conditions, and that mutations equivalent to C379A or C379A/C404S restored the acetyltransferase activity. The authors therefore proposed that the dual‐cysteine substitution present in *Bs*GlmU may inhibit activity through the formation of an intramolecular disulfide bond between C379 and C404 of the same GlmU monomer that impedes the acetyltransferase reaction. Despite *Sa*GlmU being studied previously in the context of inhibitor development, the effect of this dual‐cysteine substitution on the structure and function of the acetyltransferase domain was not considered, and how the enzyme is affected by different redox conditions (i.e., non‐reducing vs. reducing conditions) also remains unknown (Buurman et al., 2011; Green et al., 2012; Jia et al., 2023). This is important to address given that this could have direct implications for acetyltransferase inhibitor identification and may also be relevant to the biological function of *Sa*GlmU, since the enzyme will likely be exposed to different redox conditions in an infection environment (Gaupp et al., 2012). Here, we report the use of enzyme activity assays, mass spectrometry, x‐ray crystallography, and in silico modeling to characterize the functional and structural effects of the C379 and C404 variation in *Sa*GlmU and discuss the implications of our findings.

## 2 RESULTS AND DISCUSSION

Initially, the acetyltransferase activity of WT *Sa*GlmU was measured under reducing (0.5 mM DTT) and non‐reducing (no DTT) conditions to determine if this activity is redox‐sensitive. To do so, a coupled, colorimetric antimony phosphomolybdate endpoint assay was used as outlined in Figure S1. This assay is compatible with both reducing and non‐reducing conditions and provides an indirect measure of *Sa*GlmU acetyltransferase activity through coupling to the formation of pyrophosphate by the uridyltransferase activity of *Sa*GlmU.

First, a concentration range of WT *Sa*GlmU was assayed under reducing and non‐reducing conditions. Under reducing conditions, WT *Sa*GlmU was highly active, with acetyltransferase activity detected for enzyme concentrations as low as 1 nM. In contrast, under non‐reducing conditions, ~20‐fold higher concentrations of WT *Sa*GlmU were required to yield equivalent activity, supporting that the acetyltransferase activity of *Sa*GlmU is redox‐sensitive (Figure 1b). The experiment was then repeated with a C379A *Sa*GlmU mutant which, in principle, would be unable to facilitate disulfide formation with C404, as proposed previously by Wang et al. (2017) (Figure 1b). Compared to WT *Sa*GlmU, this mutant was insensitive to the redox conditions and displayed strong catalytic activity in both non‐reducing and reducing conditions, with only a small ~2‐fold increase in activity observed in the presence of DTT for equivalent concentrations of C379A *Sa*GlmU. This supports that the redox‐sensitive nature of the *Sa*GlmU acetyltransferase activity is mediated directly through C379.

Next, the effect of redox conditions on *Sa*GlmU activity was interrogated more thoroughly. First, the uridyltransferase activity of WT *Sa*GlmU was similarly measured under reducing and non‐reducing conditions by omitting the Δ251 *Sa*GlmU from the coupled assay, to determine if this activity is also redox‐sensitive. The uridyltransferase activity of WT *Sa*GlmU was unaffected by the redox conditions, confirming that the redox sensitivity is exclusive to the acetyltransferase function of *Sa*GlmU (Figure 1c). Next, the identity of the reducing agent was varied to determine which is most effective at protecting WT *Sa*GlmU activity. Surprisingly, using either 0.5 mM TCEP or 0.5 mM BME as the reducing agent in place of 0.5 mM DTT was ineffective at protecting WT *Sa*GlmU activity (Figure 1d). We speculated that DTT was more effective than BME at protecting *Sa*GlmU activity as it is a stronger reducing agent and is unable to form thiol adducts that may inhibit the acetyltransferase activity, which might be formed by BME. To test this hypothesis, intact mass measurements of untreated *Sa*GlmU or following treatment with BME or DTT were performed by ESI‐MS (Figure S2). This revealed that BME does form a single adduct with *Sa*GlmU, including during the protein purification process, but this adduct is not formed at C379 as it is still present in the C379A mutant. Given that the high acetyltransferase activity of C379A *Sa*GlmU was preserved under non‐reducing conditions, this suggests that the weaker reducing potential of BME may explain its inability to protect WT *Sa*GlmU acetyltransferase activity, rather than due to formation of BME adducts. Additionally, we suspect that DTT is more effective than TCEP as the latter is larger and may be unable to effectively access C379/C404 within the narrow acetyltransferase active site due to steric hindrance (Olsen et al., 2007; Sulzenbacher et al., 2001). The superiority of DTT over TCEP in reducing such protein disulfides has been reported previously (Cline et al., 2004). The time‐dependent inactivation of WT *Sa*GlmU was also investigated by comparing the activity of reactions where WT *Sa*GlmU was preincubated with DTT and then diluted in buffer with or without DTT (Figure 1d). This revealed that the redox‐sensitive switch is rapidly reversed (<20 min) when DTT is absent, indicating that a reducing environment is required to protect the activity of WT *Sa*GlmU.

The effect of redox conditions on the kinetics of the acetyltransferase activity was then determined by measuring the kinetic parameters for the AcCoA and GlcN 1‐P substrates in the presence and absence of 0.5 mM DTT (Figure 1e and Figure 1f). For both substrates the *k*_cat_ values under reducing conditions (*k*_cat_ ~ 60 s^−1^) were ~ 15‐fold higher than in non‐reducing conditions (*k*_cat_ ~ 3.8 s^−1^), consistent with the preliminary experiment detailed in Figure 1b. Under reducing conditions, the *K*_M_ values for AcCoA and GlcN 1‐P were determined as 0.28 ± 0.03 mM and 0.56 ± 0.1 mM, comparable to the *K*_M_ values for previously characterized GlmU isozymes (Gehring et al., 1996; Jagtap et al., 2012; Wang et al., 2017). However, under non‐reducing conditions the *K*_M_ value of AcCoA was elevated ~3‐fold to 0.82 ± 0.1 mM, while the *K*_M_ value of GlcN 1‐P was unaffected (*K*_M_ = 0.54 ± 0.1 mM). This functional analysis confirms that the acetyltransferase activity of *Sa*GlmU is regulated by a reversible redox‐sensitive switch involving C379, specifically through affecting the ability of the enzyme to utilize the AcCoA substrate.

**FIGURE 2:**
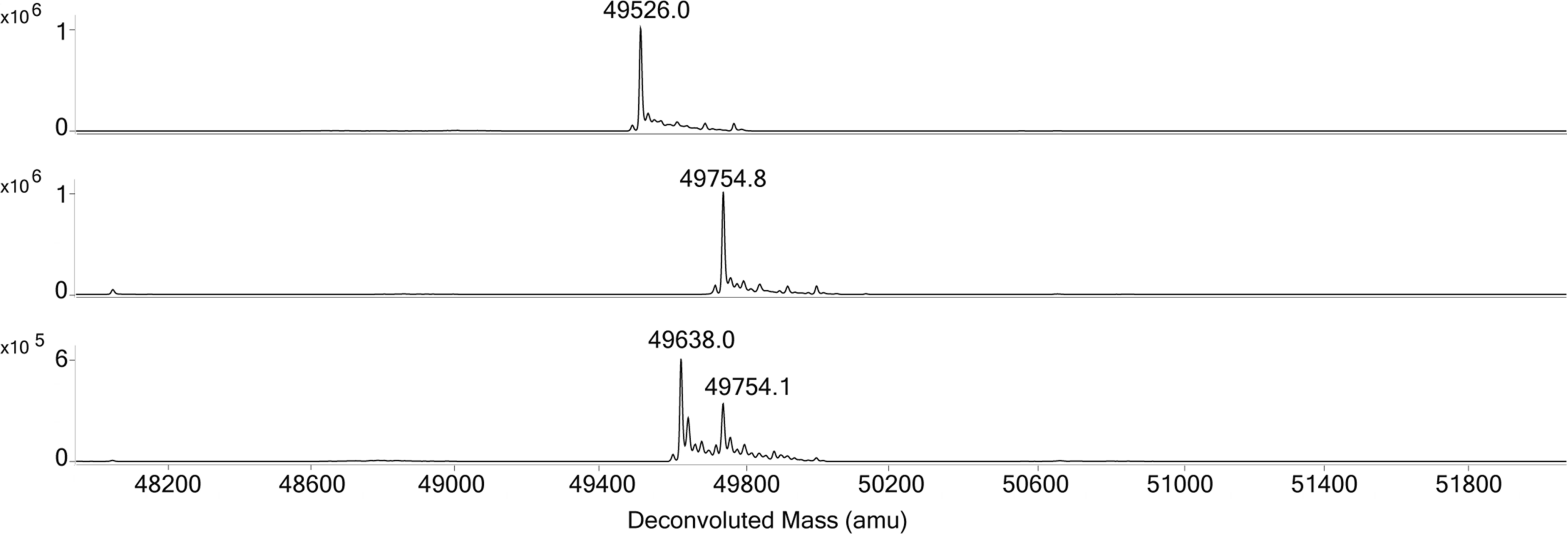
Deconvoluted mass spectra of WT *Sa*GlmU + DTT (top) WT *Sa*GlmU + guanidine‐HCl + IAA + DTT (middle) and WT *Sa*GlmU + guanidine‐HCl + IAA (bottom).

Given the results of this functional analysis, iodoacetamide (IAA) alkylation of WT *Sa*GlmU was performed in the presence and absence of DTT under denaturing conditions, and the samples analyzed by ESI‐MS to investigate whether the predicted intramolecular disulfide may be present in the enzyme (Figure 2). Under these conditions, IAA selectively reacts with any free cysteines of *Sa*GlmU (four cysteines in total), resulting in an increase in protein mass of 57 Da for each alkylated cysteine. However, oxidized cysteines, such as those forming a disulfide, will remain unmodified. The spectrum for WT *Sa*GlmU following DTT treatment (WT *Sa*GlmU + DTT) under non‐denaturing conditions revealed that the reduced protein corresponds to a single species which matches the mass of WT *Sa*GlmU with the N‐terminal methionine removed (mass = 49526.0 Da). The sample incubated with DTT prior to IAA treatment (WT *Sa*GlmU + guanidine‐HCl + IAA + DTT) yielded a single peak with a mass of 49754.8 Da, corresponding to *Sa*GlmU derivatized with four IAA adducts. In contrast, the sample of WT *Sa*GlmU alkylated under non‐reducing conditions (WT *Sa*GlmU + guanidine‐HCl + IAA) was predominantly observed as a peak with mass 49638.0 Da, corresponding to *Sa*GlmU derivatized with two IAA adducts, with a smaller proportion forming four IAA adducts (mass = 49754.1 Da). This confirms that under non‐reducing conditions the majority of WT *Sa*GlmU contains two protected cysteine residues, consistent with the formation of an intramolecular disulfide between C379 and C404.

Subsequently, x‐ray crystallography was employed to further uncover the molecular basis of the redox‐sensitive switch and how it might modulate the acetyltransferase activity of *Sa*GlmU. Crystallization experiments resulted in the determination of two cocrystal structures of WT *Sa*GlmU: a complex with AcCoA and a citrate molecule from the crystallization reagent, with the latter occupying the GlcN 1‐P site (PDB: 9DQF), and a second structure complexed with UTP, CoA, and α‐D‐glucose 1‐phosphate (Glc 1‐P) (PDB: 9DR4), a non‐reactive analogue of the GlcN 1‐P substrate. The overall fold was highly conserved in both structures, with an all‐atom RMSD of 0.76 Å. Both structures were obtained under reducing conditions in the presence of 0.5 mM DTT. While crystallization experiments were also performed with DTT omitted, no crystals with sufficient diffraction for structure determination were obtained. Data collection and refinement statistics for each structure are provided in Table S1, with electron density maps for the cocrystallized ligands presented in Figure S3.

**FIGURE 3:**
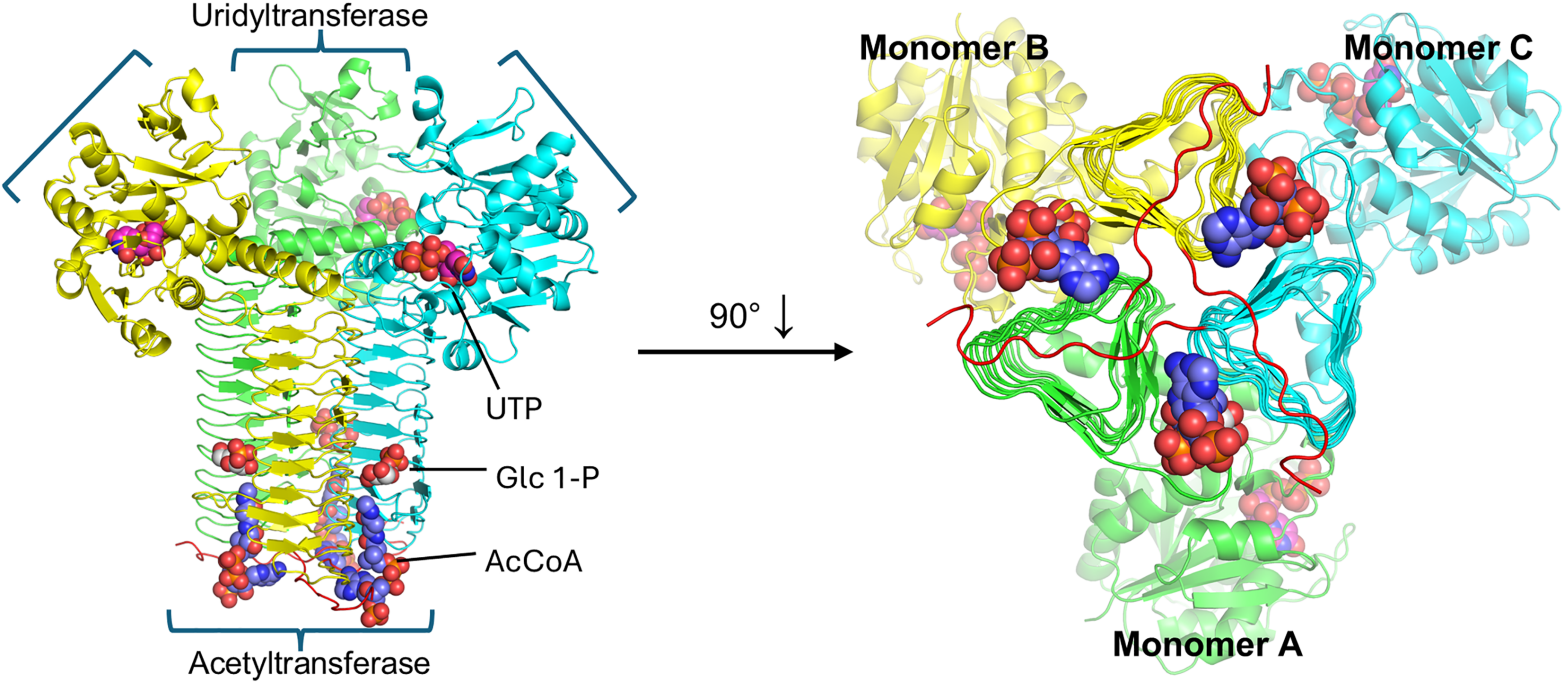
Overall structure of the *Sa*GlmU trimeric assembly. Each monomer is colored separately with the C‐terminal tail in red. The bound UTP, AcCoA and Glc 1‐P ligands are shown as pink, purple and gray spheres, respectively. The C‐terminal tail of each monomer is shown in red.

The overall structure of *Sa*GlmU was typical of other GlmU enzymes characterized to date. The *Sa*GlmU monomer present in the asymmetric unit includes two distinct domains, the N‐terminal, globular uridyltransferase domain (Res. 1–228) and the C‐terminal acetyltransferase domain (Res. 252–450) which is formed by the prominent left‐handed beta helix (L*β*H) fold, with the two domains connected by an α‐helical linker (Res. 229–251) (Figure 3). The biological trimer assembly is captured in both structures and the oligomerization interface is largely comprised of the L*β*H of the acetyltransferase domain. While the separate N‐terminal uridyltransferase domains within the trimer remain independent, the active center of the C‐terminal acetyltransferase active center is formed by components of all three monomers.

Structural comparison of the *Sa*GlmU uridyltransferase and acetyltransferase domains with the corresponding regions of *S. pneumoniae* GlmU (*Sp*GlmU; PDB: 1HM8) revealed a high degree of structural conservation, evident by the all atom RMSDs of ~0.5 Å when superimposed against both *Sa*GlmU structures (PDBs: 9DQF and 9DR4). A full amino acid sequence alignment is provided in Figure S4 for reference. Within the UTP, AcCoA, and GlcN 1‐P binding sites, the interacting residues were largely conserved between both enzymes, with only several substitutions present (Figures S5 and S6). Although a slight difference in the orientation of the captured AcCoA substrate was evident for *Sp*GlmU, all hydrogen bond interactions with the 3′‐phosphorylated ADP moiety were conserved with those of *Sa*GlmU, including interactions mediated by the C‐terminal tail residues K445 and Y448 (Figure S6A). While some substitutions are present in the hydrophobic residues stacking upon the adenine base and pantetheine group of AcCoA, all are conservative changes and facilitate similar interactions. Interestingly, due to *Sa*GlmU possessing a shorter C‐terminal tail than other GlmU isozymes, no interaction between the C‐terminal tail and the insertion loop (Res. 385–392), which forms the interface of the AcCoA and GlcN 1‐P sites, was present (Figure S6B, C). Despite this, the structure of the insertion loop matched that of *Sp*GlmU, indicating that interaction with the C‐terminal tail is dispensable for proper folding of this region and has minimal influence on the structure of the AcCoA and GlcN 1‐P binding sites in *Sa*GlmU.

**FIGURE 4:**
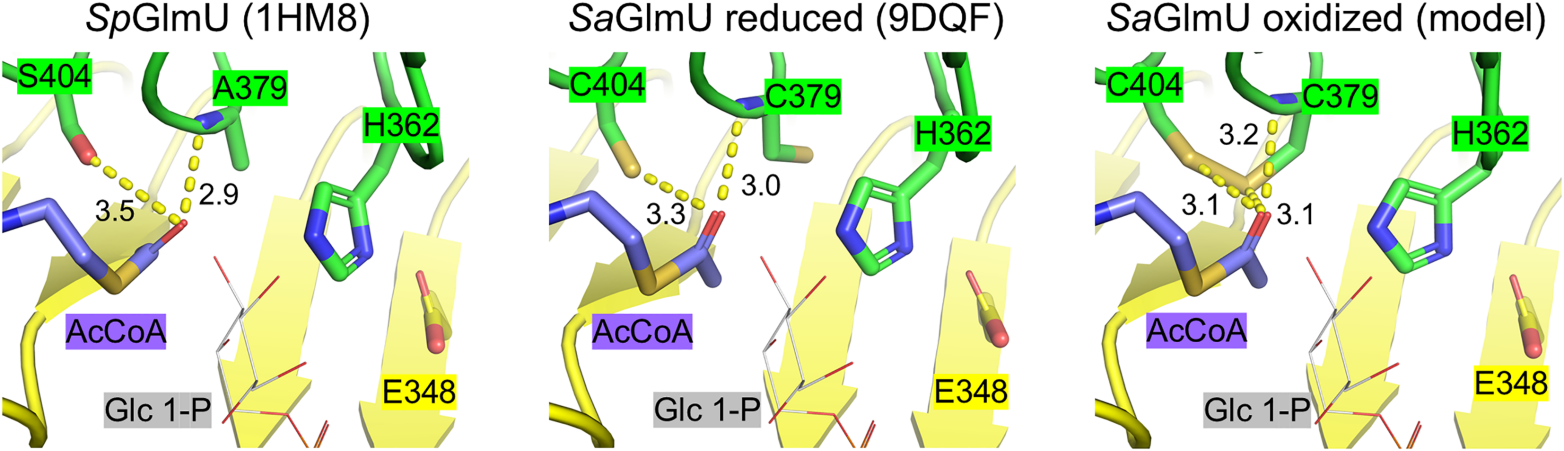
Structural comparison of the active center for (a) *Sp*GlmU (PDB: 1HM8), (b) *Sa*GlmU under reducing conditions (PDB: 9DQF, and (c) *Sa*GlmU under oxidizing conditions (in silico model). Monomer A is shown in green and Monomer B is shown in yellow. Sidechains of interest are shown as sticks of the corresponding color. The captured Glc 1‐P from PDB: 9DRF is superimposed in each panel and shown in gray as wire representation to indicate the relative location of the GlcN 1‐P site. In the right panel, the AcCoA captured in PDB: 9DQF (purple sticks) is superimposed to show the relative distance to the modeled disulfide bond. Key interactions are shown as yellow dashes with distances labeled in Angstroms.

Finally, the interface between the AcCoA and GlcN 1‐P sites of *Sa*GlmU, where C379 and C404 are located, was compared to the corresponding region of *Sp*GlmU. For the cocrystal structure of *Sa*GlmU complexed with AcCoA and citrate, which was crystallized in the presence of DTT, this region closely resembled that of *Sp*GlmU complexed with AcCoA (Figure 4). Position 379 differs between the two isozymes, with the additional thiol sidechain of C379 (*Sa*GlmU) oriented away from the acetyl group. However, this does not introduce any notable structural differences compared to *Sp*GlmU, with the sidechains of key catalytic residues E348 and H362 occupying the same positions in both enzymes (Sulzenbacher et al., 2001). C404 (*Sa*GlmU) and S404 (*Sp*GlmU) adopt the same orientation, both within hydrogen bonding distance to the thioester carbonyl of AcCoA, which is further stabilized by an additional hydrogen bond with the backbone amide of C379 (*Sa*GlmU) or A379 (*Sp*GlmU). Therefore, C404 is likely to share a similar role to S404 in the acetyltransferase reaction, which was proposed to facilitate recognition of AcCoA and/or stabilization of the resulting tetrahedral reaction intermediate (Sulzenbacher et al., 2001).

As the structure of *Sa*GlmU could not be obtained under non‐reducing conditions, a disulfide between C379 and C404 was simulated through in silico modeling of the *Sa*GlmU complex with AcCoA and citrate (PDB: 9DQF) in ICM Pro (Figure 4). To prepare this model, the crystal structure was first stripped of waters and ligands, and the geometry was optimized using the “regularization” protocol in the ICM‐Pro suite to ensure optimal geometry. The disulfide between C379 and C404 was then added, and the model was submitted to cartesian energy minimization. The resulting model predicts that C379 and C404 are in close enough proximity to form a disulfide bond (2.07 Å), accommodated by C404 adopting an alternate rotamer conformation. This is consistent with the model proposed by Wang et al. for *Bs*GlmU. Superimposing this model against the cocrystal structure of *Sa*GlmU complexed with AcCoA and citrate (PDB: 9DQF) revealed that the disulfide is in close proximity (~ 3 Å) to the AcCoA captured in the cocrystal structure, indicating it would likely clash with the substrate. Therefore, we propose that the acetyltransferase activity of *Sa*GlmU is repressed under non‐reducing conditions due to disulfide formation between C379 and C404, which simultaneously clashes with AcCoA and prevents C404 from hydrogen bonding to the thioester carbonyl of AcCoA and the subsequent tetrahedral reaction intermediate.

The discovery that the acetyltransferase activity of *Sa*GlmU is redox‐sensitive has important implications for understanding the biological function of GlmU enzymes and their inhibition. The biological relevance of this redox‐sensitive switch remains uncertain. We speculate that it may serve as a regulatory mechanism to limit UDP‐GlcNAc biosynthesis through reducing the acetyltransferase activity of *Sa*GlmU, especially on exposure to oxidative stress, but this is paradoxical in nature as UDP‐GlcNAc is required for cell wall biosynthesis. On the other hand, perhaps this may be linked to the maintenance or development of dormancy in *S. aureus*, which is induced by exposure to host‐cell oxidative stress during infection and leads to reduced translation and metabolism (Peyrusson et al., 2022). Therefore, further investigation into the functional effects of the C379/C404 dual substitution *in bacterio* is necessary. A multiple sequence alignment and subsequent phylogenetic analysis of GlmU sequences from the UniProtKB/SwissProt database revealed that the dual‐cysteine variation is only present in a small niche of Gram‐positive bacteria, being highly conserved in bacteria of the order Bacillales, including *Staphylococcus* and *Listeria*, and in *Clostridium* sp., indicating that this potential regulatory mechanism is uncommon (Figure S7). In the context of *S. aureus*, given that the cytoplasm is a reducing environment, we expect that *Sa*GlmU possesses high activity under physiological conditions in the absence of environmental stress. In contrast, we suspect that the inhibited form of *Sa*GlmU may become more abundant on exposure to external oxidative stress, which occurs in an infection environment, by altering the reducing potential of the cytoplasm and facilitating disulfide formation between C379 and C404 (Deng et al., 2013; Gaupp et al., 2012). Therefore, both states of *Sa*GlmU appear relevant for inhibitor development. However, until now, the acetyltransferase activity of *Sa*GlmU has only been assayed under non‐reducing conditions using the common 5, 5′‐dithiobis‐(2‐nitrobenzoic acid (DTNB)) colorimetric assay, which is incompatible with reducing conditions. Given that we identified functional differences for *Sa*GlmU in both turnover number and *K*_M_ for AcCoA between reducing and non‐reducing conditions, and potentially a subtle structural difference on C379/C404 disulfide formation, it is plausible that inhibitors that compete with AcCoA will display altered behavior under each condition. Therefore, we recommend that future studies for the discovery and characterization of *Sa*GlmU acetyltransferase inhibitors investigate the inhibitory activity under both reducing and non‐reducing conditions.

## 3 MATERIALS AND METHODS

### 3.1 Materials

Oligonucleotides for cloning and inverse PCR mutagenesis were purchased from Sigma (St. Louis, MO, USA). For enzyme activity assays, the substrates (UTP (U6750), GlcNAc 1‐P (A2142), GlcN 1‐P (G9753) and AcCoA (A2056)), and the components forming the color reagent (CR) (ammonium molybdate tetrahydrate (09878), potassium antimonyl tartrate trihydrate (383376), sodium dodecyl sulfate (SDS) (L4509) and L‐ascorbic acid (A5960)) were purchased from Sigma (St. Louis, MO, USA). A 2 U/μL solution of thermostable inorganic pyrophosphatase (TIPP; M0296) was purchased from New England Biolabs. The reducing agents dithiothreitol (DTT; BIMB1015), tris(2‐carboxyethyl)phosphine (TCEP; PG82080) and *β*‐mercaptoethanol (BME; 63,689) were purchased from Apollo Scientific (Bredbury, UK), Thermo Fisher Scientific (Waltham, MA, USA) and Sigma (St. Louis, MO, USA), respectively. Potassium phosphate dibasic (26931.263) and *tetra*‐sodium pyrophosphate (10261) were purchased from BDH Chemicals. For crystallization experiments, Glc 1‐P (G7000), CoA (C4780) and lithium citrate tribasic tetrahydrate (62484) were purchased from Sigma (St. Louis, MO, USA). PEG 3350 was purchased from Hampton Research (Aliso Viejo, CA, USA).

### 3.2 Gene isolation, cloning, and generation of mutants

The *glmU* gene was amplified by PCR from *S. aureus* NCTC 8325 genomic DNA, with the primers incorporating an N‐terminal hexahistidine tag, a 5′ NcoI site and a 3′ BamHI site. The resulting PCR product was ligated into the pET‐16b expression plasmid between the NcoI and BamHI restriction endonuclease sites to give the plasmid 6xHis‐*Sa*GlmU‐pET‐16b. *Sa*GlmU Δ251 and *Sa*GlmU C379A mutants were generated by inverse PCR mutagenesis as previously described, using 6xHis‐*Sa*GlmU‐pET16b as the template (Pederick et al., 2022). Oligonucleotide sequences are provided in Table S2.

### 3.3 Expression and purification of *Sa*GlmU

The constructed pET‐16b expression plasmids encoding WT *Sa*GlmU, C379A *Sa*GlmU, and Δ251 *Sa*GlmU were transformed into *E. coli* BL21(DE3) for recombinant expression. Two flasks containing 1 L of LB media supplemented with 100 μg/mL ampicillin were inoculated with 10 mL of the corresponding overnight culture. Cells were grown at 37°C to an A_600_ of 0.4–1.0 and induced with 500 μM IPTG, and *Sa*GlmU was expressed for 16 h at 16°C. Cells were then pelleted by centrifugation at 5000 × *g* for 20 min, resuspended in 15 mL of Buffer A (20 mM Tris–HCl pH 8.0, 300 mM NaCl, 10 mM imidazole, and 2 mM BME) and lysed by five rounds of cell disruption. The lysate was clarified by centrifugation at 40,000 × *g* for 20 min. All subsequent purification steps were performed at 4°C.

For enzyme activity assays, WT *Sa*GlmU was purified by nickel affinity chromatography using the batch method. The cell lysate was first incubated with 4 mL of NiNTA agarose resin (Qiagen, Venlo, Netherlands) for 1 h. The resin was washed once each with 10 mL Buffer A and 10 mL Buffer B (20 mM Tris–HCl pH 8.0, 300 mM NaCl, 25 mM imidazole and 2 mM BME). WT *Sa*GlmU was then eluted with 6× 6 mL washes of Buffer C (20 mM Tris–HCl pH 8.0, 300 mM NaCl, 150 mM imidazole and 2 mM BME). C379A *Sa*GlmU and Δ251 *Sa*GlmU were purified by nickel affinity chromatography using a 5 mL HisTrap HP column (GE Healthcare, Chicago, IL, USA) connected to an NGC Medium‐Pressure Liquid Chromatography System (Bio‐Rad, Hercules, CA, USA). Following application of the cell lysate, weakly bound impurities were removed by washing with 5 column volumes of Buffer B. Elution was carried out using a 10%–100% gradient of Buffer D over 10 column volumes (100% = 20 mM Tris–HCl pH 8.0, 300 mM NaCl, 250 mM imidazole and 2 mM BME). Fractions containing the target protein were identified by SDS‐PAGE, pooled, and dialyzed overnight against 4 L of Buffer E (20 mM Tris–HCl (pH 8.0), 100 mM NaCl and 5% glycerol). Following dialysis, the buffer was further exchanged using an Amicon Ultra centrifugal filter (30,000 molecular weight cut off; Sigma, St. Louis, MO, USA) to ensure >200‐fold dilution of BME was achieved. After buffer exchange was completed, the same centrifugal filter was used to concentrate the protein samples to 15 mg/mL (WT *Sa*GlmU and C379A *Sa*GlmU) and 30 mg/mL (Δ251 *Sa*GlmU).

For crystallization experiments, WT *Sa*GlmU was first purified by nickel affinity chromatography exactly as described for C379A *Sa*GlmU and Δ251 *Sa*GlmU. Following this, fractions containing WT *Sa*GlmU were identified by SDS‐PAGE, pooled, and concentrated using an Amicon Ultra centrifugal filter (30,000 molecular weight cut off; Sigma, St. Louis, MO, USA) and applied to a HiPrep 26/60 Sephacryl S‐200 HR gel filtration column (GE Life Sciences, Chicago, IL, USA) equilibrated with Buffer F (50 mM Tris–HCl pH 7.5, 50 mM NaCl and 2 mM DTT). Eluted fractions containing pure WT *Sa*GlmU were concentrated to 14 mg/mL and stored at −80°C. Protein concentration was determined by measuring absorbance at 280 nm using the protein molecular weight and molar extinction coefficients of 19,370 M^−1^·cm^−1^ (WT *Sa*GlmU and C379A *Sa*GlmU) and 11,920 M^−1^·cm^−1^ (Δ251 *Sa*GlmU), as calculated by ProtParam (Gasteiger et al., 2005).

### 3.4 Measurement of *Sa*GlmU activity in the presence and absence of reducing agents

To measure the acetyltransferase and uridyltransferase activities of *Sa*GlmU in the presence and absence of reducing agents, the previously described colorimetric antimony phosphomolybdate assay was used with slight modification (Pederick & Bruning, 2021). A schematic of the assay for measuring the acetyltransferase and uridyltransferase activities of *Sa*GlmU is outlined in Figure S1. All reactions were performed at 37°C in 96 well half area microplates (3695; Corning, Steuben County, NY, USA) with a final volume of 30 μL. For measuring the acetyltransferase activity, reactions contained final concentrations of 50 mM Tris–HCl pH 7.5, 10 mM MgCl_2_, 500 μM AcCoA, 500 μM GlcN 1‐P, 200 μM UTP, 0.01 U/μL TIPP, 500 nM Δ251 GlmU, and varying concentrations of WT *Sa*GlmU or C379A *Sa*GlmU. For measuring the uridyltransferase activity, reactions contained 50 mM Tris–HCl pH 7.5, 10 mM MgCl_2_, 200 μM UTP, 200 μM GlcNAc 1‐P, 0.01 U/μL TIPP, and varying concentrations of WT *Sa*GlmU or C379A *Sa*GlmU. In both cases, WT *Sa*GlmU or C379A *Sa*GlmU was prepared as a 3× stock solution in 50 mM Tris–HCl pH 7.5 ± 1.5 mM reducing agent (therefore giving 0.5 mM final concentration of reducing agent in each reaction) and 10 μL of the *Sa*GlmU stock solution was added as the final component to initiate the reaction. For the preincubation experiment, *Sa*GlmU was first diluted to a concentration of 20 μM in 50 mM Tris–HCl pH 8 + 1.5 mM DTT and incubated on ice for 10 min. Following the incubation, the sample was diluted to 6 nM in either 50 mM Tris–HCl pH 7.5 or 50 mM Tris–HCl pH 7.5 + 1.5 mM DTT, and then 10 μL of each diluted sample was used to initiate separate reactions. Reactions were allowed to proceed for exactly 10 min, with the reactions quenched by the addition of 120 μL 1.25× CR (159 mM sulfuric acid, 0.75 mM ammonium molybdate, 0.3125% w/v SDS, 11.25 mM L‐ascorbic acid and 45 mM potassium antimonyl tartrate) kept at room temperature. Following the addition of 1.25× CR, the plate was immediately moved to room temperature and color development was allowed to proceed for 10 min, after which the absorbance was read at 850 nm using a Pherastar FS microplate reader (BMG Labtech, Ortenberg, Germany). To account for acid‐catalyzed UTP hydrolysis during color development, the absorbance of a no enzyme “blank” reaction was subtracted from the corresponding sample. *Sa*GlmU activity was quantified using the slope of a sodium pyrophosphate standard curve generated in the presence of either acetyltransferase or uridyltransferase substrates (Figure S8). All experiments were performed three times, with a single sample measured per experiment for each condition assayed.

### 3.5 Determination of kinetic parameters for *Sa*GlmU acetyltransferase activity

The kinetic parameters of the *Sa*GlmU acetyltransferase activity were also determined using the antimony phosphomolybdate assay with the same general procedure. The reaction buffer consisted of 50 mM Tris–HCl pH 7.5, 10 mM MgCl_2_, 200 μM UTP, 0.01 U/μL TIPP, 500 nM Δ251 *Sa*GlmU, 2 nM WT *Sa*GlmU (0.5 mM DTT) or 40 nM WT *Sa*GlmU (no DTT), with the concentrations of AcCoA and GlcN 1‐P varied. To determine *K*_M_ values, either AcCoA or GlcN 1‐P was held constant at a high concentration of 3 or 2 mM, respectively, while the other substrate was varied. The varied substrate was assayed at seven concentrations ranging between at least 0.2× and ~4× the measured *K*_M_. All *K*_M_ values were derived by fitting data with the Michaelis–Menten Equation (Equation (1)) by nonlinear regression in GraphPad Prism 9 (San Diego, CA, USA). All experiments were performed three times, with a single sample measured per experiment for each condition assayed.

(1) $$v=\frac{v_{\max}\left[S\right]}{K_{M}+\left[S\right]}.$$

### 3.6 Determination of protein mass by mass spectrometry

For non‐alkylated samples, 100 μL of 15 mg/mL WT *Sa*GlmU or C379A *Sa*GlmU was diluted 10‐fold with 100 mM ammonium bicarbonate pH 7.9 and incubated at 22°C for 30 min. For the WT *Sa*GlmU samples prepared under reducing conditions, the buffer also contained either 10 mM BME or 5 mM DTT. Following incubation, the samples were buffer exchanged >4000‐fold into 100 mM ammonium bicarbonate pH 7.9 using an Amicon Ultra centrifugal filter (10,000 molecular weight cut off; Sigma, St. Louis, MO, USA) and then concentrated to a final concentration between 3 and 5 mg/mL for analysis. For selective alkylation of WT *Sa*GlmU under non‐reducing and reducing conditions, 200 μL samples were prepared containing 3.75 mg/mL WT *Sa*GlmU, 6 M guanidine‐HCl, and 100 mM ammonium bicarbonate pH 7.9 ± 5 mM DTT and incubated at 37°C for 30 min to facilitate protein denaturation. Iodoacetamide was then added to a final concentration of 15 mM and the samples incubated in the dark for 30 min at 22°C. Samples were then buffer exchanged >10,000 000‐fold into 100 mM ammonium bicarbonate pH 7.9 using an Amicon Ultra centrifugal filter (10,000 molecular weight cut off; Sigma, St. Louis, MO, USA) to remove residual guanidine HCl and then concentrated to a final concentration of ~2 mg/mL for analysis. Protein mass measurements were carried out under denaturing conditions using an Agilent 6230 time‐of‐flight instrument coupled to an Agilent 1260 Infinity II LC System. Protein sample (3 μL) was injected and electrosprayed using 50% aqueous acetonitrile/0.01% formic acid at a flow rate of 0.2 mL/min, without chromatographic separation. ESI‐MS conditions were: positive‐ion mode; capillary voltage, 3500 V; nozzle voltage, 2000 V; fragmentor, 175 V; gas, 8 L/min; gas temperature, 325°C; sheath gas, 11 L/min; and sheath gas temperature, 350°C; *m*/*z* range, 500–3200. Spectra were deconvoluted using the Maximum Entropy algorithm in BioConfirm software (Agilent), over the mass range 48,000–52,000 Da.

### 3.7 Crystallization of *Sa*GlmU

Conditions facilitating crystallization of *Sa*GlmU were identified using the PEG‐Ion HT sparse matrix screen (Hampton Research). Screening was completed by the sitting drop vapor diffusion method in 96 well Intelliplates (Art Robbins, Sunnyvale, CA, USA). Each well contained 80 μL of crystallization reagent, with 2 μL drops containing 1 μL of reservoir solution and 1 μL of *Sa*GlmU in the presence or absence of 3 mM AcCoA. This identified condition D9 (200 mM lithium citrate tribasic tetrahydrate, 20% PEG 3350) as a candidate for further optimization, and this condition was ultimately used to obtain diffracting crystals. For crystallization of *Sa*GlmU in complex with AcCoA and citrate, 14 mg/mL *Sa*GlmU was incubated with 2.5 mM AcCoA for 30 min on ice. For crystallization of *Sa*GlmU in complex with UTP, CoA, and Glc 1‐P, 14 mg/mL *Sa*GlmU was incubated with 1 mM CoA, 3.3 mM UTP, 3.3 mM MgCl_2_, and 3.3 mM Glc 1‐P for 30 min on ice. Diffracting crystals were obtained by the hanging drop vapor diffusion method in 24 well plates (Costar, Corning) containing 500 μL of reservoir solution (200 mM lithium citrate tribasic tetrahydrate, 15%–20% PEG 3350), with drops containing 1 μL *Sa*GlmU and 1 μL of reservoir solution. Crystals appeared after several days and grew to full size within 1–2 weeks.

### 3.8 Data collection, structure determination, model refinement, and in silico modeling

Prior to data collection crystals were transferred to Paratone‐N for cryoprotection and flash‐frozen in liquid nitrogen (Teng, 1990). Diffraction images (360° at 0.1° oscillation) were collected at the MX2 beamline of the Australian Synchrotron (Aragão et al., 2018; Broennimann et al., 2006). Indexing and integration were completed using XDS (Kabsch, 2010). Scaling and merging of datasets were completed using Aimless (CCP4) (Winn et al., 2011). The *Sa*GlmU complex with AcCoA and citrate (PDB: 9DQF) was solved by molecular replacement in Phaser, using separate N‐terminal (Res. 12–257) and C‐terminal (Res. 259–460) components of *S*. *pnuemoniae* GlmU (PDB: 1HM8) as the search models, prepared using CHAINSAW (McCoy et al., 2007; Stein, 2008). To phase the *Sa*GlmU complex with UTP, CoA, Glc 1‐P (PDB: 9DR4), the R‐free flags of the former isomorphous *Sa*GlmU model were first transferred to the corresponding dataset using FREERFLAG (CCP4). Modeled ligands and waters were removed from all search models. Solutions from Phaser were refined in Phenix. These models were subjected to multiple rounds of rebuilding in Coot, followed by B‐factor and refinement in Phenix until refinements converged (Adams et al., 2010; Emsley & Cowtan, 2004). Data collection and refinement statistics are provided in Table S1. Electron density maps for cocrystallized ligands are presented in Figure S3. In silico modeling of the C379/C404 disulfide bond was performed in ICM Pro (Molsoft L. L. C., San Diego, CA, USA) using the cocrystal structure of *Sa*GlmU complexed with AcCoA and citrate (PDB: 9DQF). The crystal structure was first stripped of waters and ligands and then prepared using the “regularization” protocol to ensure optimal geometry of the model. The disulfide bond between C379 and C404 was then modeled using the “Set Disulfide Bond” option. The resulting model was submitted to 5000 steps of cartesian energy minimization using default settings prior to analysis. Alignment of enzyme structures by secondary structure matching and structure visualization was performed using PyMol (The PyMol Molecular Graphics System, Version 2.3.0., Schrödinger, LLC, New York, NY, USA).

### 3.9 Phylogenetic analysis of GlmU isozyme protein sequences

For comparative sequence analysis, a protein blast search against the UniProtKB/Swiss‐Prot database was performed, identifying ~1,000 sequences with homology to LdmS. Sequences were filtered to exclude those with <80% sequence coverage. Redundancy was reduced through manual curation, and the retained sequences were aligned in MEGA‐X using MUSCLE with default parameters (377 entries in total) (Kumar et al., 2018). These aligned representative sequences were used as the input for phylogenetic analysis in IQ‐TREE2 for construction of a maximum‐likelihood tree using the LG + R10 substitution model (identified using ModelFinder) and tested using 10,000 ultrafast bootstrap replicates (Hoang et al., 2018; Kalyaanamoorthy et al., 2017; Minh et al., 2020). The generated tree was visualized as an unrooted tree using iTOL (Figure S6) (Letunic & Bork, 2016).

## AUTHOR CONTRIBUTIONS

**Jordan L. Pederick:** Conceptualization; investigation; writing – original draft; methodology; validation; visualization; formal analysis; data curation; writing – review and editing. **Akhil Kumar:** Investigation; methodology; writing – review and editing. **Tara L. Pukala:** Investigation; methodology; visualization; writing – review and editing. **John B. Bruning:** Project administration; supervision; resources; writing – review and editing.

## CONFLICT OF INTEREST STATEMENT

The authors declare no conflicts of interest.

## Supporting information


**Data S1:** Supporting Information

**Data S2:** Supporting Information

## Data Availability

The data that support the findings of this study are available from the corresponding author upon reasonable request.
