# Supplementary material for: Functional and structural characterization of Staphylococcus aureus N‐acetylglucosamine 1‐phosphate uridyltransferase (GlmU) reveals a redox‐sensitive acetyltransferase activity
Source: Protein Sci. 2025 Mar 27;34(4):e70111. doi: 10.1002/pro.70111 (PMC11947611; doi:10.1002/pro.70111)
Supplement: Supplementary file 1 — Data S1: Supporting Information [file PRO-34-e70111-s002.pdf]

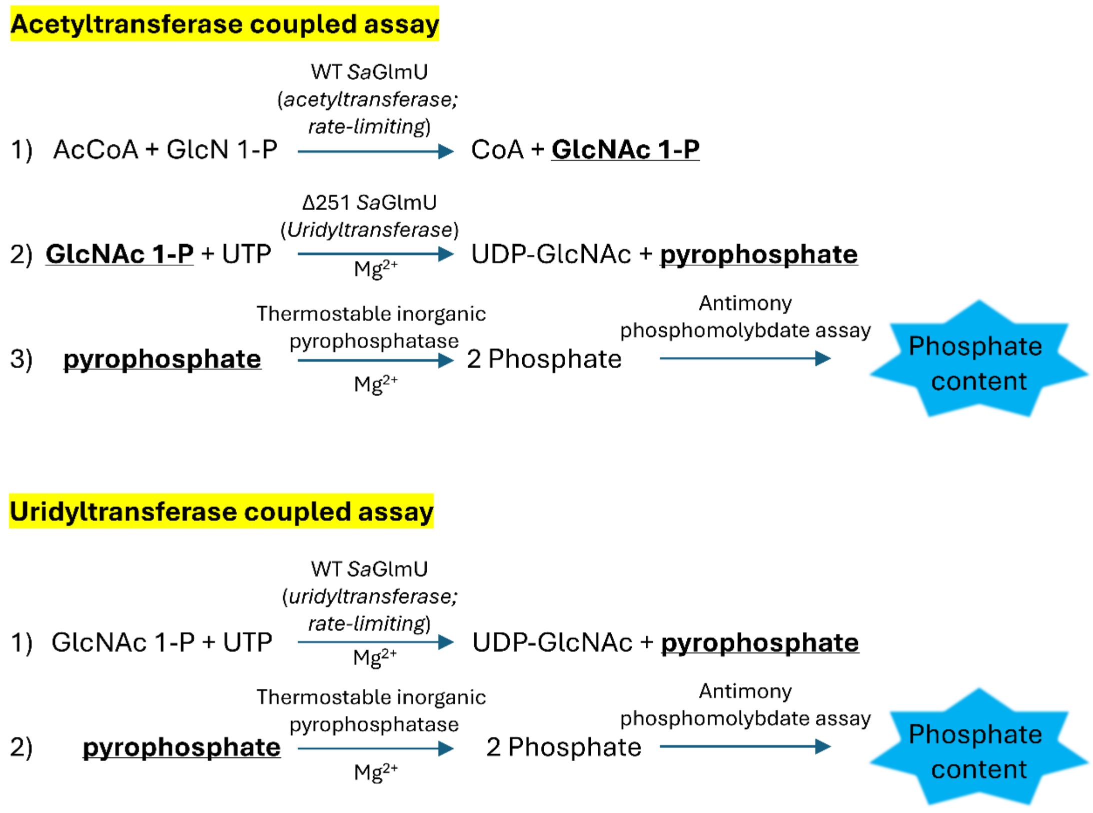

Figure S1. Application of the antimony phosphomolybdate assay for monitoring the acetyltransferase and uridyltransferase activities of *Sa*GlmU. The bolded and underlined substrates/products indicate those which are used in the subsequent reaction. For the acetyltransferase coupled assay, GlcNAc 1-P production is monitored through coupling to the uridyltransferase activity of the *Sa*GlmU N-terminal domain (Δ251 *Sa*GlmU). This results in the formation of pyrophosphate which can be liberated to phosphate through the activity of thermostable inorganic pyrophosphatase (TIPP). The phosphate content of the sample can then be quantified using the antimony phosphomolybdate assay to determine the concentration of GlcNAc 1-P determined during the reaction, with the GlcNAc 1-P content = 0.5× phosphate content. In the uridyltransferase coupled pyrophosphate production by WT *Sa*GlmU is monitored through coupling to the activity of TIPP, producing phosphate. The phosphate content is determined by the same method as the acetyltransferase coupled assay, with the pyrophosphate concentration = 0.5× phosphate content assay the same concept applies except that the uridyltransferase activity of WT *Sa*GlmU measured.

Table S1. Crystallographic data collection, processing and refinement statistics.

Statistic	*Sa*GlmU AcCoA + citrate PDB: 9DQF	*Sa*GlmU UTP, CoA, Glc 1-P PDB: 9DR4
**Wavelength (Å)**	0.9537	0.9537
**Resolution range (Å)**	35.82 -1.9 (1.97 -1.9)	39.21 - 1.85 (1.92 - 1.85)
**Space group**	H 3 2	H 3 2
**Unit cell**		
a, b, c (Å)	95.98 95.98 277.83	94.88 94.88 262.53
α, β, γ (°)	90 90 120	90 90 120
**Total reflections**	792586 (81549)	796494 (80221)
**Unique reflections**	39293 (3890)	39284 (3885)
**Multiplicity**	20.2 (21.0)	20.3 (20.6)
**Completeness (%)**	99.75 (99.90)	99.87 (99.92)
**Mean I/sigma(I)**	13.59 (1.45)	12.14 (1.25)
**Wilson B-factor**	31.81	24.05
**R-merge**	0.144 (2.49)	0.239 (3.67)
**R-meas**	0.148 (2.55)	0.246 (3.76)
**R-pim**	0.0329 (0.555)	0.0543 (0.824)
**CC1/2**	0.999 (0.703)	0.999 (0.561)
**Reflections used in refinement**	39213 (3886)	39244 (3883)
**Reflections used for R-free**	1984 (197)	1996 (210)
**R-work**	0.198 (0.286)	0.175 (0.285)
**R-free**	0.232 (0.323)	0.213 (0.310)
**Number of non-hydrogen atoms**	3567	3703
macromolecules	3313	3349
ligands	64	92
solvent	190	262
**Protein residues**	449	450
**RMS (bonds)**	0.008	0.009
**RMS (angles)**	0.92	1.03
**Ramachandran favored (%)**	98.43	98.88
**Ramachandran allowed (%)**	1.57	1.12
**Ramachandran outliers (%)**	0	0
**Rotamer outliers (%)**	1.15	2.26
**Clashscore**	3.47	2.96
**Average B-factor**	42.70	30.46
macromolecules	42.37	29.52
ligands	48.81	41.46
solvent	46.45	38.57

[a] Closed brackets contain values for the highest resolution shell.

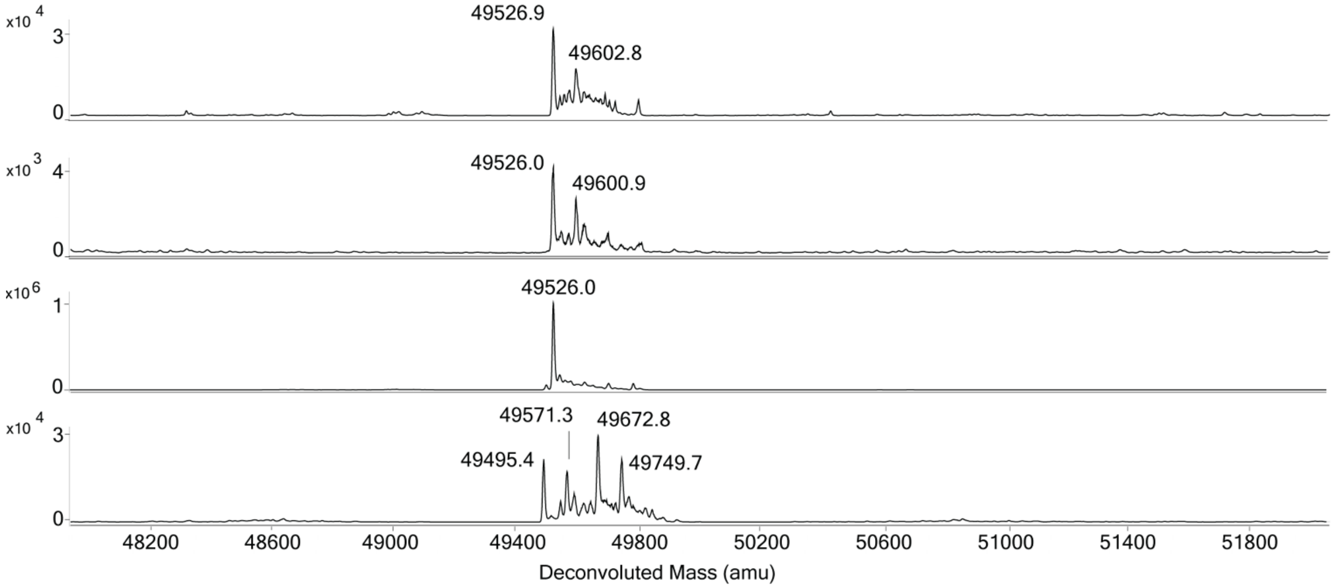

Figure S2. Deconvoluted mass spectra of untreated WT *Sa*GlmU (top), BME-treated WT *Sa*GlmU (upper middle), DTT-treated WT *Sa*GlmU (lower middle; reproduced from Figure 2) and untreated C379A *Sa*GlmU (bottom). For WT *Sa*GlmU the peaks with mass ~ 49 526 correspond to the protein with the N-terminal methionine removed, while the peaks with mass ~ 49 601 correspond to the BME adduct. For C379A *Sa*GlmU, the peaks with mass 49 495.4 and 49 571.3 correspond to the protein with the N-terminal methionine removed, and the same species with the BME adduct, respectively. The peaks with mass 49 672.8 and 49 749.7 correspond to the protein with an intact N-terminal formylmethionine, and the same species with the BME adduct, respectively.

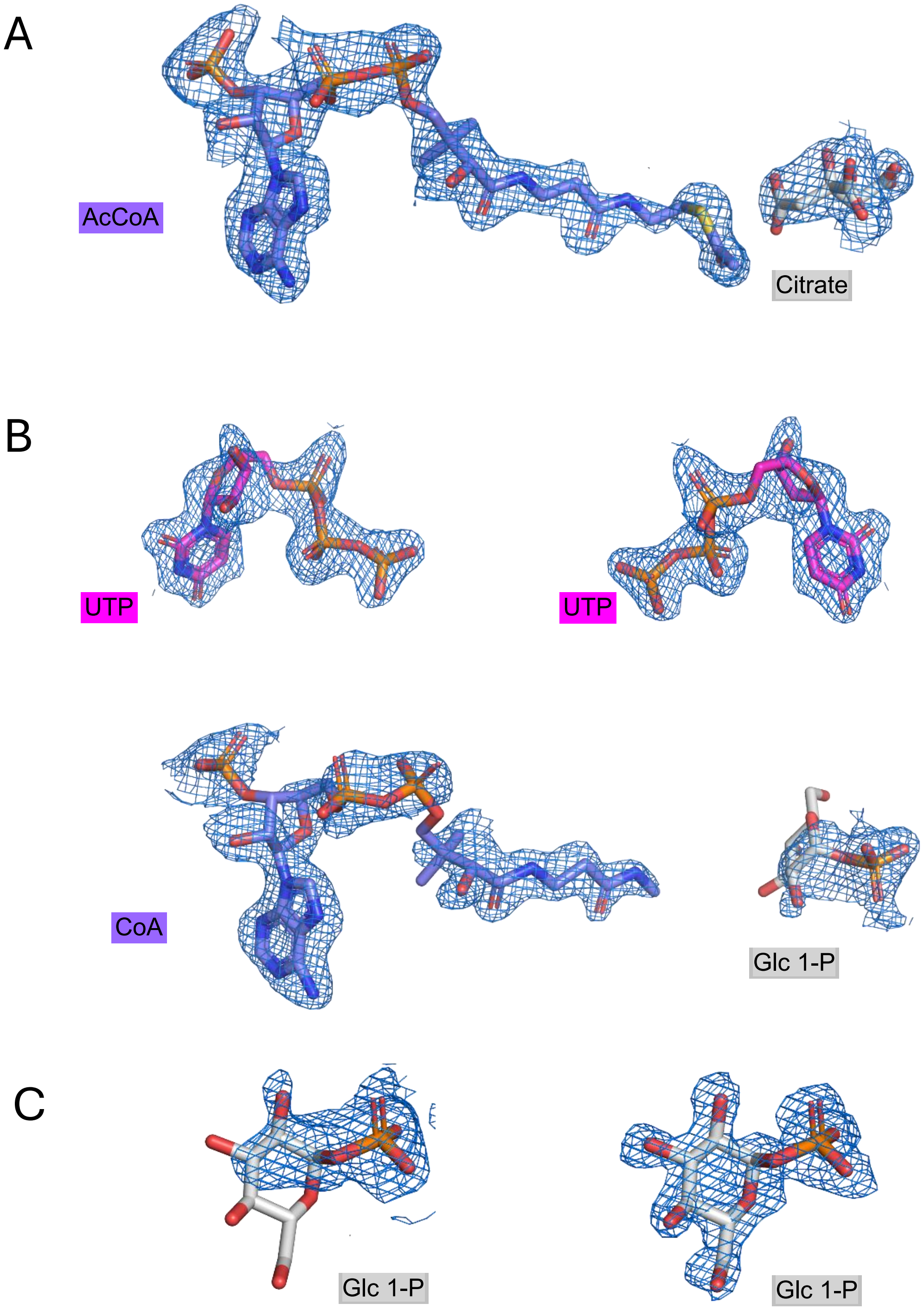

Figure S3. Electron density maps (2Fo-Fc; 1σ) for captured ligands. **A)** Simulated annealing composite omit electron density for AcCoA and citrate in PDB: 9DQF. **B)** Simulated annealing composite omit electron density for UTP, CoA and Glc 1-P in PDB: 9DR4. Part of the cysteamine group of CoA (CH2-SH) was not modeled as no electron density for these atoms was observed. **C)** Close-up view of simulated annealing composite omit electron density (left) and feature enhanced electron density (right) for Glc 1-P.

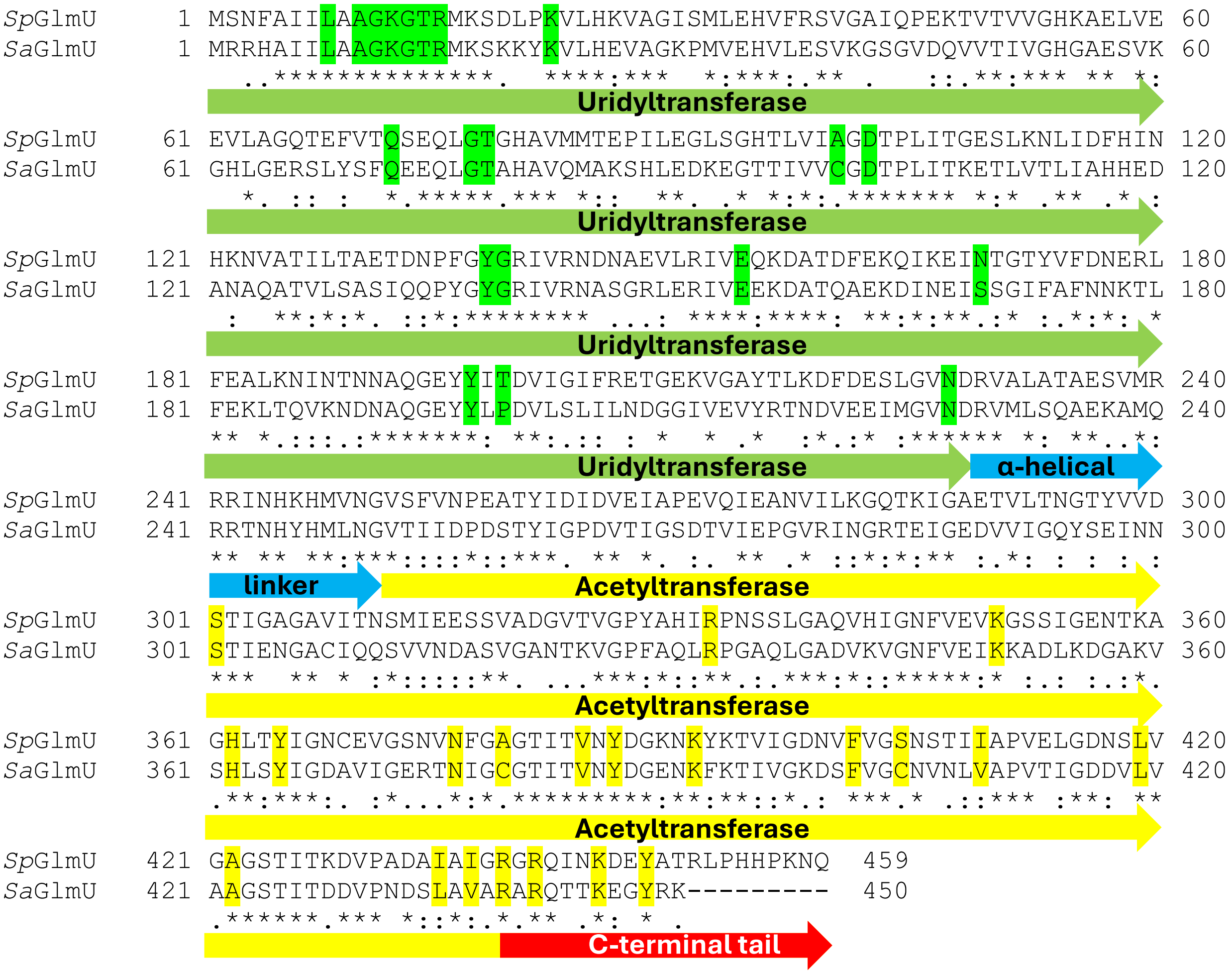

Figure S4. Amino acid sequence alignment of *Sp*GlmU and *Sa*GlmU isozymes. The uridyltransferase domain, α-helical linker, acetyltransferase domain and C-terminal tail are labeled. Amino acids involved in substrate recognition within the uridyltransferase, and acetyltransferase domain are colored green and yellow, respectively. ***** = positions with a single, fully conserved residue; **:** = positions with conservation between amino acid groups of similar properties; **.** = positions with conservation between amino acid groups of weakly similar properties.

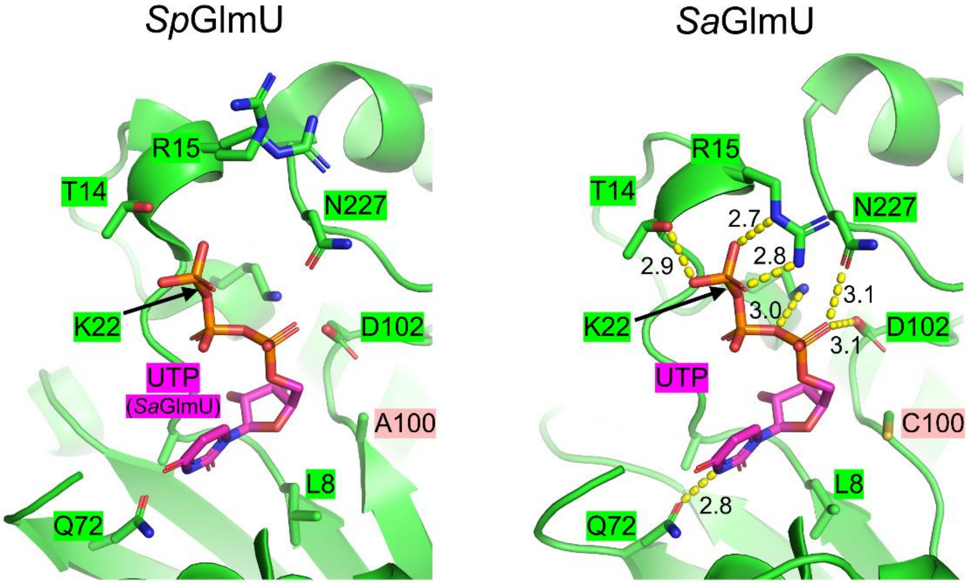

Figure S5. Comparison of UTP binding within the uridyltransferase domain of *Sp*GlmU (left; 1HM8) and *Sa*GlmU (right; 9DR4). Interacting residues conserved between the two isozymes are labeled in green. Interacting residues that differ between the two enzymes are labeled in pale pink. The UTP captured in PDB: 9DR4 is shown as pink sticks and is superimposed against *Sp*GlmU for reference. Yellow dashes indicate hydrogen bond interactions, with distances labeled in angstroms.

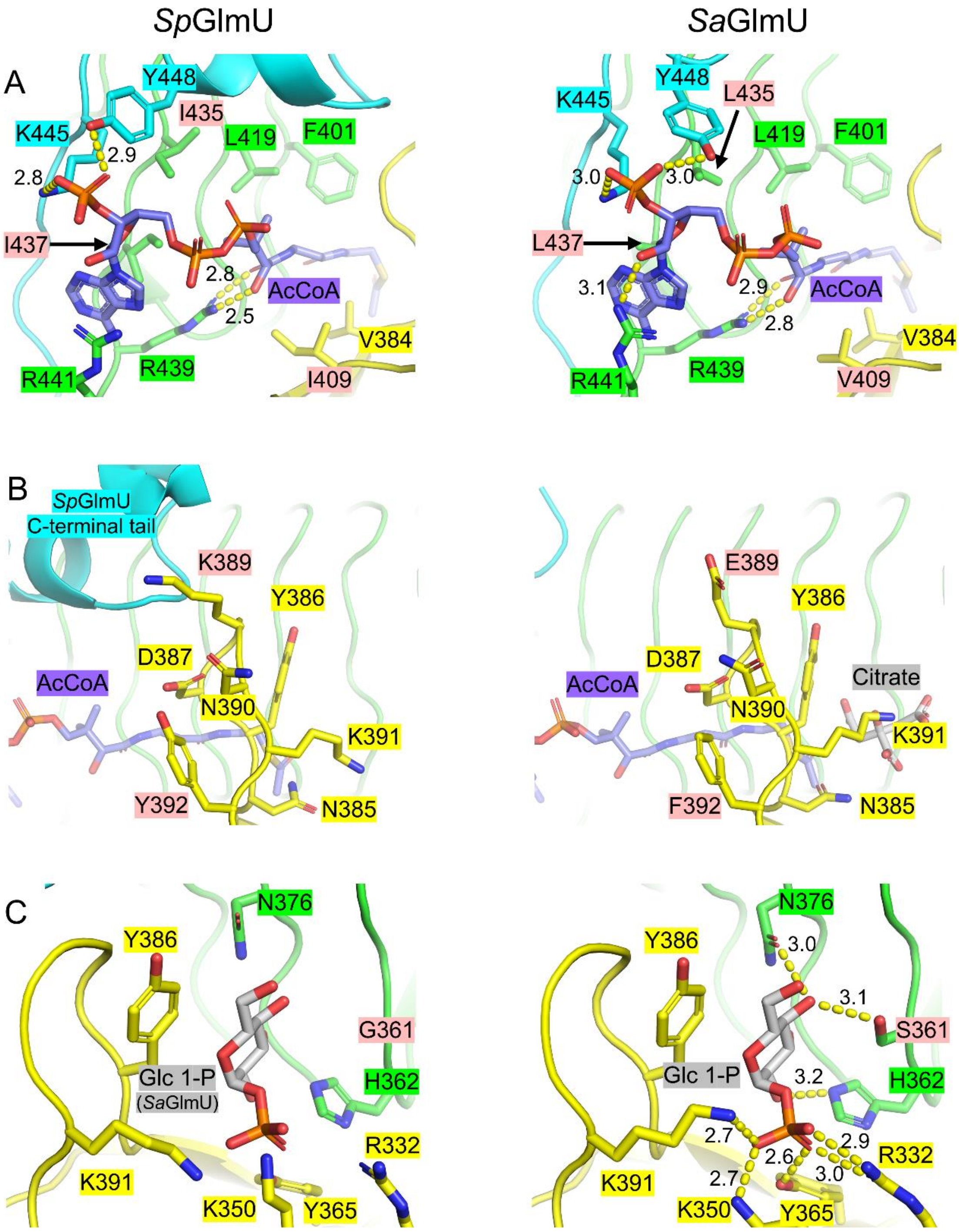

Figure S6. Comparison of ligand binding within the acetyltransferase domain of *Sp*GlmU (left; 1HM8) and *Sa*GlmU (right; 9DR4). **A)** Comparison of AcCoA binding site, **B)** Comparison of insertion loop structure, and **C)** Comparison of GlcN 1-P binding site. Monomers A, B and C of each enzyme are shown in green, yellow and cyan, respectively. Interacting residues conserved between the two isozymes are labeled in the corresponding color. Interacting residues that differ between the two enzymes are labeled in pale pink. AcCoA is shown as purple sticks, with citrate and Glc 1-P shown as gray sticks. In panel C), the Glc 1-P captured in PDB: 9DR4 is superimposed against *Sp*GlmU for reference. Yellow dashes indicate hydrogen bond interactions, with distances labeled in angstroms.

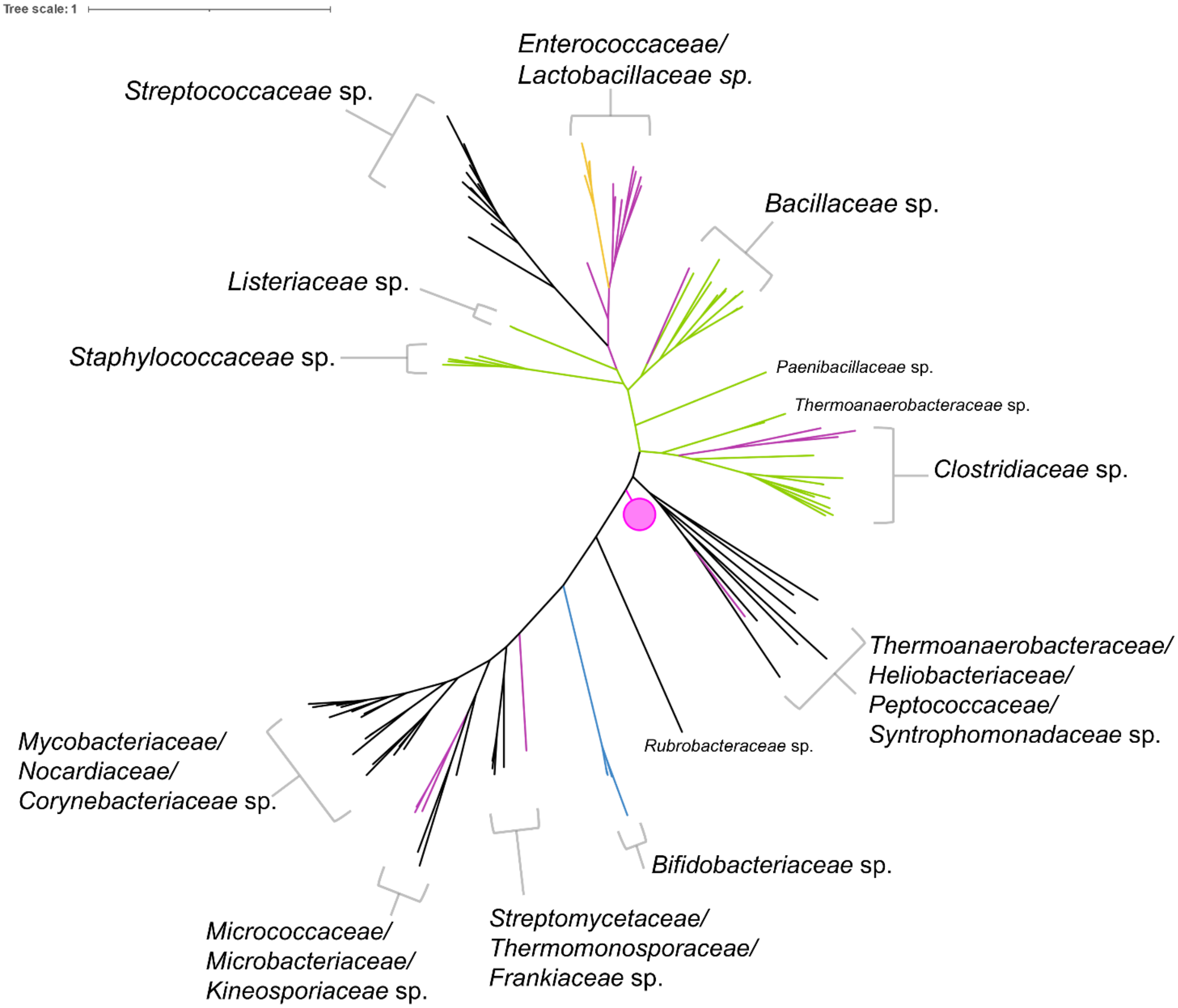

**Variant type (positions 379/404)**	**# in Firmicutes** (105 entries)	**# in other phyla** (271 entries)
A/S	40	223
C/C	34*	1
C/S	17	19
C/A	5	0
G/A	5	0
A/A	2	17
A/T	1	5
A/I	1	0
C/I	0	5
G/S	0	1
A/V	0	1

Figure S7. Unrooted phylogenetic tree of curated GlmU protein sequence alignment. The coloring of each branch in the phylum Firmicutes indicates the identity of the residues corresponding to Positions 379/404 in *S. aureus*. Entries corresponding to other phyla are collapsed into a clade, colored in bright pink. Green: C/C, Purple: C/S, Yellow: C/A, Blue: G/A, Black; A/S. Distances for each displayed branch are to scale. All observed variants are summarized in the included table. * One entry in the phylum Firmicutes which possessed the C/C variation is within the collapsed clade.

Table S2. Oligonucleotides used for cloning of WT *Sa*GlmU and generation of *Sa*GlmU mutants by inverse PCR mutagenesis.

Oligonucleotide name	Sequence (5' → 3')[a, b, c]
WT *Sa*GlmU F	AAT*CCATGG*GC**CATCACCATCACCATCAC**GGCAGCATGCGAAGACACGCG
WT *Sa*GlmU R	ATT*GGATCC*TTATTTCCTATATCCTTCTTTTGT
*Sa*GlmU Δ251 F	TAATAAGTGACAATCATCGATCCTG
*Sa*GlmU Δ251 R	ACCATTTAGCATGTGATAATG
*Sa*GlmU C379A F	TAATATTGGTGCGGGAACGATTACAGTTAACTATG
*Sa*GlmU C379A R	GTACGTTCGCCAATTACAG

[a] Restriction endonuclease recognition sites are italicised and underlined.
[b] The sequence encoding the hexahistidine tag is shown in bold and underlined.
[c] Substitutions producing the *Sa*GlmU Δ251 and C379A mutations are indicated by green highlighting.

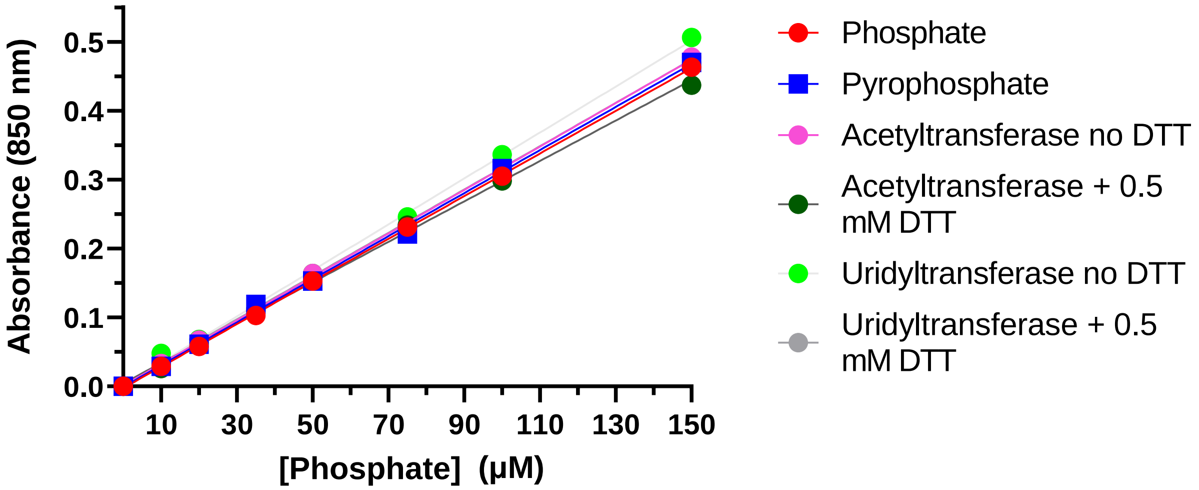

Reaction conditions	Slope
Phosphate	0.00310 ± 0.00002
Pyrophosphate	0.00312 ± 0.00005
Acetyltransferase conditions no DTT	0.00315 ± 0.00004
Acetyltransferase conditions + 0.5 mM DTT	0.00293 ± 0.00005
Uridyltransferase conditions no DTT	0.00333 ± 0.00005
Uridyltransferase conditions + 0.5 mM DTT	0.00314 ± 0.00004

Figure S8. Phosphate and pyrophosphate standard curves generated in the presence and absence of DTT for the acetyltransferase reaction conditions and uridyltransferase reaction conditions. All samples were incubated at 37°C for 10 min prior to color development. The phosphate standard curve was generated in 20 mM Tris-HCl pH 7.5. The pyrophosphate standard curve was generated in buffer containing 20 mM Tris-HCl pH 7.5, 10 mM $MgCl_2$ and 0.01 U/μl TIPP. Notably, the slopes of these standard curves are the same, indicating that TIPP is present in sufficient excess to convert all pyrophosphate to phosphate over the course of the 10 min reaction period. Acetyltransferase conditions included 20 mM Tris-HCl pH 7.5, 10 mM $MgCl_2$, 3 mM AcCoA, 1 mM GlcN 1-P, 200 μM UTP, 0.01 U/μl TIPP and 500 nM Δ251 *Sa*GlmU, using pyrophosphate as the phosphate source. Uridyltransferase conditions included 20 mM Tris-HCl pH 7.5, 10 mM $MgCl_2$, 200 μM GlcNAc 1-P 200 μM UTP, and 0.01 U/μl TIPP, using pyrophosphate as the phosphate source. Under these conditions 0.5 mM DTT was found to have a minimal effect on the slope of the standard curve generated using pyrophosphate, indicating that the selected conditions are compatible with both assays.
